# Supplementary material for: BioDiscViz: A visualization support and consensus signature selector for BioDiscML results
Source: PLoS One. 2023 Nov 30;18(11):e0294750. doi: 10.1371/journal.pone.0294750 (PMC10688618; doi:10.1371/journal.pone.0294750)
Supplement: Attachment — Submitted filename: Response to Reviewers.docx [file pone.0294750.s002.docx]

# Response to reviewers

**Reviewer #1: This is not an original research paper, but rather a technical note of a visualisation extension of an already exiting software. I do not think it fits the scope of this journal and therefore, rejection is recommended**

While we respect the opinion of the reviewer, our manuscript fits the scope of PLOS ONE. According to the stated criteria, a tool must be useful to the community and demonstrate a clear advantage over existing alternatives, if applicable. For BiodiscViz, there is currently no comparable visualization tool available for machine learning results from a software like BioDiscML where many models are computed at once. Additionally, BiodiscViz represents an original contribution that significantly enhances the interpretation of machine learning results, particularly in the field of biology where researchers often possess limited knowledge in computer science. Therefore, there is a genuine need for a tool like BiodiscViz.

**Reviewer #2: This manuscript presents BioDiscViz, an R-based visualization package for understanding and investigating the results outputted by BioDiscML. I think the tool itself is interesting and could be very useful to the users of the BioDiscML package, the presentation of the tool in this manuscript is also good but could use some improvement. For that reason, I think this manuscript is suited for publication with some editions.**

We are pleased that the reviewer agrees with our assessment that the manuscript describes a potentially very useful tool. We have carefully considered all suggestions to enhance the research paper and the accompanying tool. We are resubmitting improved versions addressing all the comments.

**1. I was somewhat confused about the layout of BioDiscViz when I read the paper. More specifically, the authors mentioned 4 main parts, I was not sure what it was referring to, and only two of them are presented in figures 1A and 1B, so I was wondering what's on the other two parts. It's probably better to show the whole UI first, introduce each part on the UI, and focus on each individual section in later parts, or at least rearrange the existing text so it's more clear.**

We thank the reviewer for pointing out this lack of clarity. We revised the Layout section and incorporated the explanation provided on GitLab to improve clarity and understanding of our tool.

**2. The authors mentioned the tool could provide an "interactive table" of the data used for the short and long signatures. I'm not quite sure what that means, but it sounds like an interesting feature. It would be great to see more details about it and probably an illustration if the authors find it fit.**

The reviewer is raising an interesting question. The interactive table was not a primary feature of our tool, so we did not delve into extensive detail in the paper. The idea was to let the user access the input table without leaving the application while giving them easier means to select and search for instance or values. We are now including a brief explanation of the table and supplemented it with illustrations in the supplementary data to provide a visual demonstration of its functionality. Furthermore, as we realized that the term wasn’t the most appropriate we will just use “table” to describe it.

**3. For a visualization tool, some of the plots are a little bit crude and hard to read. One simplest changes I would suggest is increasing the font sizes of the plots, as well as the size of the scatters on the PCA plots. It would be best to make them customizable by the users.**

The comment by the reviewer relates to simplicity versus versatility of the tool. Our primary goal was to ensure that the application is highly user-friendly by minimizing the number of interactive parameters that users need to manage. However, we acknowledge that this approach could be problematic if the plots are difficult to read. Therefore, we have implemented a reactive value for the fontsize of the plots. This allows users to adjust the fontsize at any time, addressing any readability issues and providing them with greater control over their viewing experience.

**4. Other dimension reduction plots might also be useful, like Umap, TSNE, in addition to PCA, just something to consider.**

We agree with the reviewer that additional dimension reduction plots would be useful. As such the Umap, and t-SNE are now included.

**Reviewer #3: The paper presents an application, BioDiscViz, implementing different visualization techniques and exploratory tools to investigate BioDiscML outputs, that is outputs from a software implementing different Machine Learning algorithms for the identification of biomarker signatures. While I believe that the developed application has merit and that the paper is clear and well written, I have some comments for the authors.**
We are grateful that the reviewer appreciated the merit of our application. This new version of our manuscript addresses all the concerns.

**- Currently, the application is written using the R library Shiny, but it is only hosted locally on one of the authors' gitlab pages (**[**https://gitlab.com/SBouirdene/biodiscviz**](https://gitlab.com/SBouirdene/biodiscviz)**). The Shiny environment allows for free and open access web publication of Shiny applications, via** [**https://www.shinyapps.io/**](https://www.shinyapps.io/)**. I believe the authors should make their application easily accessible on the web. In its current state, it requires for users to manually install it and run it locally in R, after having cloned the gitlab repository containing the application. This is a somehow lengthy process that could inhibit use of the application and I believe should be avoided as primary option to access it, given that a freely available platform for publishing is available. You could still leave the current option (run the app locally) as a secondary one, for more experienced users to choose.**
We thank the reviewer for the suggestion. To provide a web alternative, we now include a web option for our application to cater to the users' needs. It is now hosted in shinnyapps.io, in the free version.

**- In relation to my previous comment, multiple packages (ComplexHeatmap - which has to be manually installed from Bioconductor, Rtools - for which the corresponding drives need to be downloaded and installed manually) are required to run the app. However, the requirement of having to install such dependencies is not stated in the instructions available in the gitlab page. This results in quite a long and multiple steps installation and deployment process for a user who does not have these library already pre-installed locally. I suggest the authors improve such aspect.**

Once again, the reviewers raised an important limitation. In order to prioritize user-friendliness, we improved the tool to specifically integrate Bioconductor directly into the app. This will automatically download required packages if not already present on the user's system. Additionally, we included a reference in the readme file to guide users on downloading Rtools. Furthermore, the installation of all the libraries used in the app has been seamlessly integrated and will be automatically implemented when the user runs it. These enhancements will simplify the setup process for users and provide a smoother experience overall.

**- While the illustration of usage of the app is clear on the gitlab page and on the paper, no runnable toy example is available on the app. I believe including a toy example, which would not require the upload of a BioDiscML output, would be a very useful feature of the app, that could serve better illustrating its functioning to first time users. You could consider including the same example discussed in this paper and in the gitlab page**

Initially, we included the dataset used for the research paper as an example in the "example" directory, aiming to help users become acquainted with the tool. However, it seems like our decision created confusion. We have now implemented an "example" button, which automatically loads the data specifically for this example, ensuring a better user experience. Additionally, to provide further clarity on the functioning of the app, we included a step-by-step video guide, allowing users to follow along and better understand the app's functionality. These additions will enhance user understanding and usability of the tool.
